# A Phylogenetic and Taxonomic Revision of *Discula theae-sinensis*, the Causal Agents of Anthracnose on *Camellia sinensis*

**DOI:** 10.3390/jof10020141

**Published:** 2024-02-09

**Authors:** Meijun Guo, Shiyi Zhao, Yue Gao, Xiaoye Shen, Chenglin Hou

**Affiliations:** College of Life Science, Capital Normal University, Xisanhuanbeilu 105, Haidian, Beijing 100048, China; guoguomjun@126.com (M.G.); zsyiiiiii@163.com (S.Z.); 2220802061@cnu.edu.cn (Y.G.)

**Keywords:** tea anthracnose, *Camellia sinensis*, phylogeny, taxonomy, Koch’s postulates

## Abstract

Tea (*Camellia sinensis* (L.) Kuntze) is one of the most important economic plants in China, and has many benefits for human health. Anthracnose is one of the most serious diseases of tea in China, and control of the fungus is important since most Chinese cultivars are susceptible to it. The agent of tea anthracnose was initially described as *Gloeosporium theae-sinensis* I. Miyake in Japan, which was later transferred to *Discula*, but this taxonomic position remains problematic. To shed light on these taxonomic and phylogenetic issues, the tea anthracnose pathogens were re-studied. Combining the morphological characteristics and a multigene phylogenetic analysis of nrITS, nrLSU, *rpb2*, and *tef1* sequence data, a new genus *Sinodiscula* was proposed to accommodate the causal fungi of tea anthracnose, including a new species *Sinodiscula camellicola* and a new combination *Sinodiscula theae-sinensis*. Furthermore, the pathogenicity of the pathogens was determined according to Koch’s postulates. This study thoroughly resolves the long-standing taxonomic and phylogenetic problems of the tea anthracnose pathogens.

## 1. Introduction

Tea (*Camellia sinensis* (L.) Kuntze), as one of the most popular non-alcoholic beverages, contains various chemical ingredients that are beneficial to human health, which can effectively reduce the risk of human diseases [1,2,3,4,5,6], and is loved by consumers worldwide. At present, China is home to 18 major tea-producing provinces, with a total tea plantation area of approximately 3.3303 hectares [6,7]. In 2022, China’s total output of tea reached 3.1810 million tons, with a national total output value of CNY 318.068 billion, making it the leading producer and exporter of tea worldwide [7]. *Camellia sinensis* has emerged as one of the most crucial economic crops in southern China, particularly in mountainous regions [6,8].

Anthracnose is a highly detrimental disease affecting tea plants in both Japan and China [8,9,10], and has also been reported in Sri Lanka [11]. In Japan, tea anthracnose is regarded as one of the three major diseases affecting tea plantations, together with the tea exobasidium blight and the tea white scab [11]. Anthracnose causes extensive necrosis and abscission of tea leaves, leading to adverse effects on tea plant growth, as well as decreased yield and quality of tea [8,12].

The causal fungus of anthracnose was first described as *Gloeosporium theae-sinensis* I. Miyake (*Dilocarpon*, *Drepanopezizaceae*, *Helotiales*, *Leotiomycetidae*, *Leotiomycetes*, *Pezizomycotina*) in Japan [13]. Yamamoto [14] transferred *G. theae-sinensis* to the genus *Colletotrichum* Corda (*Glomerellaceae*, *Glomerellales*). This combination, however, was invalid because Yamamoto did not cite its basionym (ICBN, Vienna Art 33.4). Moriwaki and Sato [9] transferred *G. theae-sinensis* to *Discula* (*Gnomoniaceae*, *Diaporthales*) based on morphological and molecular data. However, *Discula* species are known as polyphyletic, and *Discula theae-sinensis* is phylogenetically distant from the type species of *Discula*; the taxonomic position of *Discula theae-sinensis* is still unclear [15,16].

In China, the pathogen of tea anthracnose was initially reported as *Gloeosporium theae-sinensis* [17], and this name continues to be used to this day [5,8,10,18]. Until 2023, the name *Discula theae-sinensis* has been adopted [18]. Recently, tea anthracnose has been on the rise in high-humidity and low-lying tea areas in Hunan, Fujian, Zhejiang, Guizhou, and other provinces, resulting in substantial economic losses for tea plantations and posing a serious threat to the tea industry [7,19,20].

The objective of this study is to revise the taxonomy and phylogenetics of the causal agent of tea anthracnose. A total of 32 strains of the pathogens of tea anthracnose were obtained from diseased leaves of the tea in Anhui, Hubei, and Zhejiang Provinces, China. Based on morphological characteristics and multigene phylogenetic analysis (nrITS, nrLSU, *rpb2*, and *tef1*), a new genus *Sinodiscula* is introduced and a novel species and a new combination are proposed. We expect our findings to provide a reference for the effective prevention and control of tea anthracnose on *Camellia sinensis*.

## 2. Materials and Methods

### 2.1. Fungal Collection and Isolation

Disease leaves of tea anthracnose were collected from the tea leaves collected in Anhui, Hubei, and Zhejiang Provinces, China. Each sample was marked and placed in kraft bags, brought back to the lab, and preserved at room temperature before being processed. The tissues at the junction of disease and health were cut into 5 mm^2^ fragments and disinfected in 75% ethanol for 30 s followed by 10% sodium hypochlorite for 5 min and washed in sterile water three times. The fragments were then placed on potato dextrose agar (PDA) and incubated at room temperature for a month. The cultures are deposited in the China Forestry Culture Collection Center (CFCC) and the Mycology Laboratory of Capital Normal University (CNUML), Beijing, China.

### 2.2. Morphological Studies

Cultures were transferred to PDA culture medium at room temperature under a dark light environment and growth rates were observed daily for a month, including colony color, texture, and the conidiomata; the cultures’ color was described according to the color charts of Rayner [21]. Spores were produced at room temperature, naturally. Cultures were examined periodically for sporulation. Conidia were taken from pycnidium and placed in sterilized water. The diseased tissues with large black small spots were picked out from the tea leaves, then 3–5 mm^2^ small fragments were cut out and placed under a stereo microscope to be cut as thin as possible. The cut fragments were placed in sterile water, and the darker part was picked for microscopic examination of the cross-section of the conidioma. Aniline blue (cotton blue) was used to stain colorless structures (conidiomata, conidiogenous cells, and conidia). The shape and size of microscopic structures were observed and noted using a light microscope (Olympus DP71, Tokyo, Japan).

### 2.3. DNA Extraction, PCR Amplification, and Sequencing

The diseased leaves with conidiomata were picked out from the dried tea leaves, then 2 mm^2^ small fragments were cut out and crushed by shaking for 45 s at 30 Hz 2–4 times (Mixer Mill MM301, Retsch, Haan, Germany) in a 1.5 mL tube together with 3 mm diam. tungsten carbide balls, and total genomic DNA was extracted using M5 Plant Genomic DNA Kit (Mei5 Bioservices Co., Ltd., Beijing, China) following the manufacturer’s instructions. Total genomic DNA was extracted from fresh mycelium that was gained by scraping the surface of 7-day-old colony on PDA, using M5 Plant Genomic DNA Kit following the manufacturer’s instructions. This result is consistent with the positive control result, which proves the accuracy of the experimental data. PCR amplification and sequencing of the LSU nrDNA region using the primer pair LROR/LR5 [22,23], ITS nrDNA region using primer pair ITS1F/ITS4 [24,25], rpb2 region using the primer pair fRPB2-5f/ fRPB2-7cR [26], and tef1 region using primer pair EF1-728f/EF2 [27,28] were performed (Table 1).

In order to accurately identify pathogenic fungi, the sequences of ITS, nrLSU, *rpb2*, and *tef1* were amplified from the type strain of *Discula theae-sinensis* (MAFF 752003) for further research.

The temperature profile for both ITS nrDNA and LSU nrDNA was an initial denaturing step for 2 min at 94 °C, followed by 35 amplification cycles of denaturation at 94 °C for 60 s, annealing at 58 °C for 60 s and extension at 72 °C for 90 s, and a final extension step of 72 °C for 10 min [29]. The temperature profile for the rpb2 was initial denaturation at 94 °C for 120 s, followed by 35 amplification cycles of denaturation at 95 °C for 45 s, annealing at 57 °C for 50 s, and extension at 72 °C for 90 s [24]. The temperature profile for tef1 was initial denaturation at 94 °C for 120 s, followed by 35 amplification cycles of denaturation at 95 °C for 30 s, 58 °C for 50 s, 72 °C 60 s [30]. PCR productions were estimated visually in agarose electrophoresis gel by comparing band intensity with a DNA ladder of 200 bp, purified and sequenced by Zhongkexilin Biotechnology Co., Ltd. (Beijing, China).

### 2.4. Phylogenetic Analyses

The new sequences were submitted to the GenBank database and additional sequences of ITS, nrLSU, *rpb2*, and *tef1* included in this study were downloaded from GenBank (Table 2). Sequences of further taxa were included, and isolates were selected to represent each of the 31 known families in the *Diaporthales* based on the latest available literature. The ITS, nrLSU, *rpb2*, and *tef1* datasets were aligned with MAFFT [31], and then manually corrected visually in Se-Al v.2.03a [32]. Ambiguously aligned regions were not used in the analyses. A combined dataset of ITS, nrLSU, *rpb2*, and *tef1* sequences was prepared and analyzed using the maximum parsimony method performed with PAUP * 4.0b10 [33]. Maximum parsimony analysis was conducted using heuristic searches with 1000 replicates of random-addition sequence, tree bisection reconnection (TBR) branch swapping, and no maxtree limit. All characteristics were equally weighted and unordered. Gaps were treated as missing data to minimize homology assumptions. A bootstrap analysis was performed with 1000 replicates, each with 100 random taxon addition sequences. Maxtree was set to 1000, and TBR branch swapping was employed. For the Bayesian analysis, MrModeltest 2.3 with the Akaike information criterion (AIC) was used to choose the substitution model for each gene: GTR + I + G for ITS, nrLSU, and *rpb2*, HKY + I + G for *tef1*. The Bayesian analysis was performed with MrBayes 3.1.2 [34,35]. The analyses of four chains were conducted for 100,000,000 generations with the default settings and sampled every 100 generations, halting the analyses at an average standard deviation of split frequencies of 0.01. The first 25% of trees were removed as burn-in. Bayesian posterior probabilities (PP) were obtained from the 50% majority rule consensus of the remaining trees. Maximum likelihood (ML) analysis was performed with IQ-TREE 2.2.0 [36], the substitution model for each gene: TIM2e + I + G4 for ITS, TIM3e + I + I + R3 for nrLSU, TN + F + I + G4 for *rpb2*, and HKY + F + I + I + R4 for *tef1*, respectively. ML bootstrap replicates (1000) were computed in IQ-TREE using a rapid bootstrap analysis and search for the best-scoring ML tree. We only considered clades supported by bootstrap values (MLB) ≥70% for the ML analysis, supported by bootstrap values (MPB) ≥ 70% for the MP analysis, and supported by PP ≥ 95% for Bayesian inference. The final alignments and the retrieved topologies were deposited in TreeBASE (http://www.treebase.org, accessed on 13 January 2024), under accession ID: 31091.

### 2.5. Pathogenicity Tests

The isolates CFCC 1914-2-2 and CFCC 107-4-1 were selected to fulfill Koch’s postulates. Healthy one-year-old seedlings of *Camellia sinensis* with a height of approximately 0.2 m were obtained from Anhui Province. Each isolate was inoculated on three separate seedlings and three leaves were selected on each seedling for inoculation. Before the pathogenicity experiment, the surfaces of the leaves were sprayed with 75% alcohol 2–3 times, and then the above operation was repeated with sterile water to remove the residual alcohol; then, they were dried with absorbent paper, or we waited for the surfaces to dry [37]. Sterilized needles (0.5 mm diam.) were used to wound five times in the middle parts of each disinfected leaf close to the margins on both sides. The 5 mm PDA medium plugs with mycelia from a 5-day-old culture were inoculated on the left side of the wounded leaves, and sterile PDA plugs without mycelia were inoculated in parallel on the right side of the wounded leaves as control, and repeated three times. A conidial suspension was also used for inoculation. When testing conidial suspensions (10^6^ per mL in sterile distilled water), 10 μL of the suspension was deposited on one side of the tested leaves using a syringe, with 10 μL of sterile water acting as control on the opposite side. However, like the results obtained by Li et al. [18], the spore suspension could not bring about obvious disease spots. Each inoculated tea plant was placed in a light incubator at 28 °C and 75% relative humidity with a 12/12 h light/dark photoperiod, and the disease progression of the leaves was regularly observed. The experiment was repeated three times. To complete Koch’s postulates, as previously mentioned, the fungi were reisolated from the margin tissue of the diseased lesions that developed from the inoculated tissue and were identified via molecular and phylogenetic analysis.

## 3. Results

### 3.1. Molecular Phylogeny

**Figure 1:**
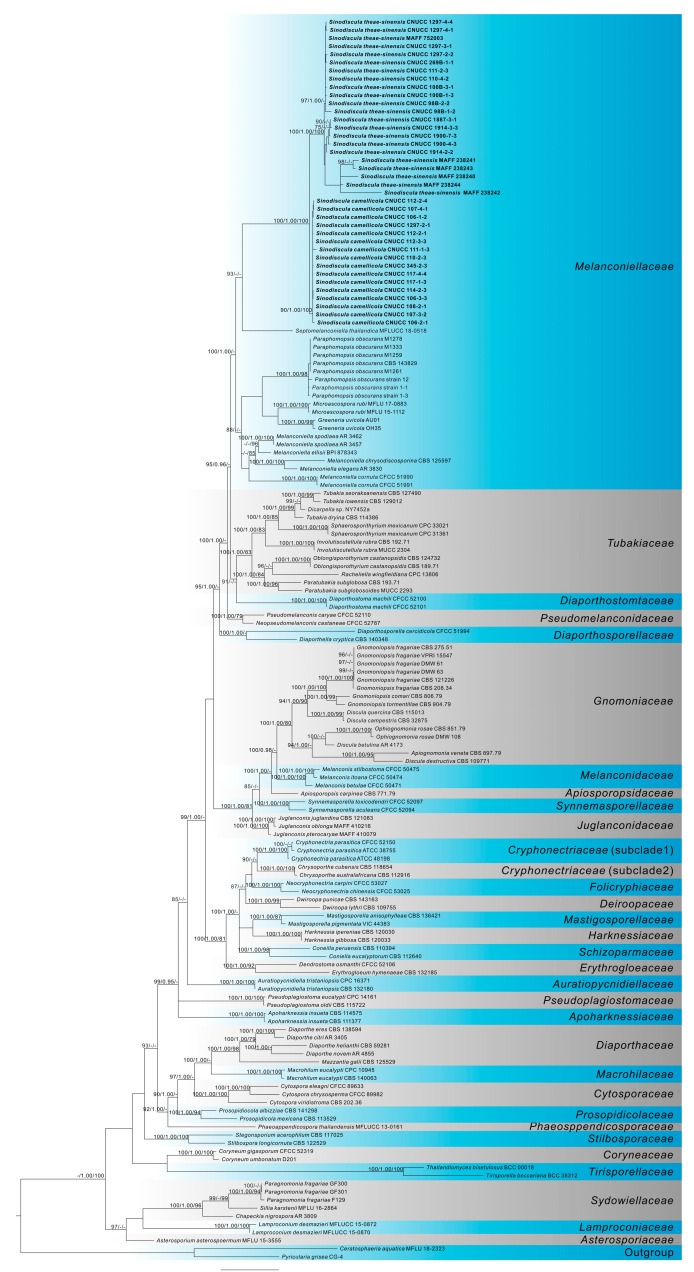
Phylogenetic tree derived from maximum likelihood analysis of the combined ITS, nrLSU, *rpb2*, and *tef1* sequences of *Diaporthales*, using *Ceratosphaeria aquatica* (MFLU 18.323) and *Pyricularia grisea* (CG4) as the outgroups. Bootstrap support values for RAxML and maximum parsimony greater than 70% and Bayesian posterior probabilities (PP) greater than 0.95 are given below and above the nodes. New species and new combinations from this study are in **bold**.

The sequence dataset of *Diaporthales* including 32 strains from this study and 114 reference strains from recent studies was analyzed based on ITS, nrLSU, *rpb2*, and *tef1* [38,39,40,41,42,43,44,45,46], and *Ceratosphaeria aquatica* Z.L. Luo, K.D. Hyde and H.Y. Su and *Pyricularia grisea* Cooke ex Sacc. as the outgroups. The multi-gene dataset (gene boundaries of ITS: 1–534, nrLSU: 535–1317, *rpb2*: 1318–2228, *tef1*: 2229–3132) comprised 3132 characters including the alignment gaps, of which 1647 were parsimony-informative, 213 parsimony-uninformative, and 1272 constant. The MP analysis of sequences resulted in one most parsimonious tree (Figure 1) with a length (TL) of 13,431 steps, consistency index (CI) of 0.280, retention index (RI) of 0.714, rescaled consistency index (RC) of 0.200, and homoplasy index (HI) of 0.720.

Based on the combined four-gene (ITS-nrLSU-*rpb2*-*tef1*) analysis, all *Diaporthales* species are supported as one clade (MLB = 100, MPB = 100, PP = 1.00). The 147 isolates clustered in 32 clades corresponding to 31 families in *Diaporthales*.

The isolates of the pathogen of tea anthracnose we obtained, clustered into a novel phylogenetic taxon within *Melanconiellaceae*, which formed a strong support clade (MPB = 100, MLB = 100, PP = 1.00) and are distinct from their closest relatives classified in *Greeneria* Scribn. and Viala, *Melanconiella* Sacc., *Microascospora* Senan. and K.D. Hyde., *Paraphomopsis* Udayanga and Castl., or *Septomelanconiella* Samarak. and K.D. Hyde. In this clade, there are two subclades with high support values, among them, the type strain of *Discula theae-sinensis* (MAFF 752003) is clustered in clade 2 (Figure 1).

### 3.2. Taxonomy

*Sinodiscula* M. J. Guo and C. L. Hou, gen. nov.—MycoBank MB851774.

*Etymology*. *sino* (lat.) = China, referring to the specimens collected in China.

Asexual morph: The front of the lesion is scattered with many black, small protruding granules that are pycnidial conidiomata. Conidiomata acervular, irregularly round or oval, erumpent to immersed, solitary, scattered. Conidiogenous layer covering the entire inner surface of acervular chambers and mostly in basal layer, yellowish-brown, initially developing under epidermis, then breaking through epidermis and forming thick whitish amorphous conidial masses. Conidiophores acropleurogenous, branched or sympodially branched, cylindrical, aseptate. Conidiogenous cells enteroblastic, phialidic, cylindrical, straight or slightly curved, crowded, terminal, slightly tapering toward apex. **Conidia** abundant, small, acrogenous, hyaline, fusiform to obovoid, often biguttulate, tapered at base or both ends.

Sexual morph: Undetermined.

Type species: *Sinodiscula theae-sinensis* (I. Miyake) M. J. Guo and C. L. Hou, described below.

The multiple-gene phylogenetic analysis shows that sequence data obtained from specimens cited below for species of *Sinodiscula* form an independent clade with high support values (MLB = 100, MPB = 100, PP = 1.00). Morphologically, *Sinodiscula* can be distinguished from its closely related genera *Greeneria*, *Melanconiella*, *Microascospora*, *Paraphomopsis*, and *Septomelanconiella*. In contrast to the new genus, the asexual morph of *Melanconiella* usually consists of septate only at the base and hyaline to light brown conidiophores, annellidic or phialidic conidiogenous cells, dark brown melanconium-like or hyaline discosporina-like conidia [46,47]. Similarly, the genus *Greeneria*, which is typified by *Greeneria uvicola* (Berk. and M.A. Curtis) Punith., forms pale brown conidia, variously shaped ranging from fusiform, oval, to ellipsoidal, each with a truncate base and obtuse to bluntly pointed apex [48]. The asexual morph of *Paraphomopsis* distinct from *Sinodiscula* by pycnidia with a slightly elongated, black neck, wider toward the apex at maturity [46]. The asexual morph of *Septomelanconiella* distinct from *Sinodiscula* by mature conidia cylindrical to clavate, straight or slightly curved, brown, 1-euseptate, more often with six unequal lumina, guttulate, dark brown at the base [49]. Although the asexual morph of *Microascospora* remains undetermined, *Microascospora* is distantly related to *Sinodiscula* in the phylogeny presented (Figure 1). Additionally, the sexual morph of *Microascospora* is distinct from other genera in the same family having immersed, solitary ascomata with narrow papilla with smaller hyaline, aseptate ascospores bearing long appendages [40,41,46].

**Figure 2:**
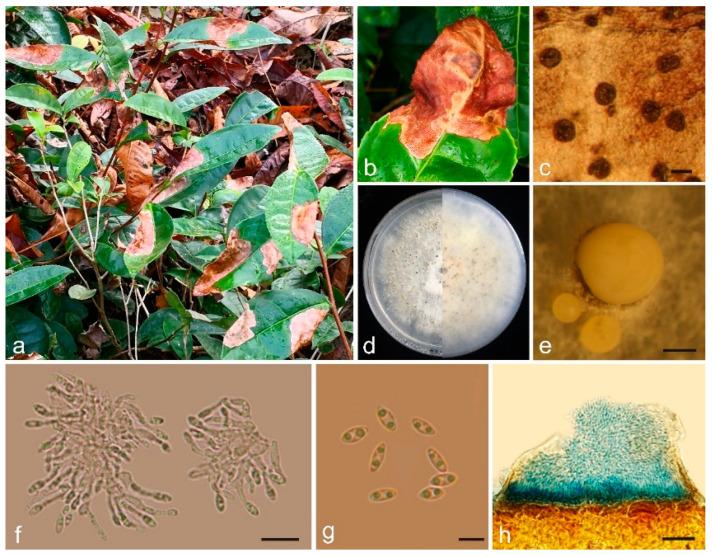
Morphology of *Sinodiscula camellicola* collected from *Camellia sinensis:* (**a**) *Sinodiscula camellicola* causes a large number of fallen leaves of *Camellia sinensis*; (**b**) Lesion on *Camellia sinensis*; (**c**) Habit of conidiomata on leaf of *Camellia sinensis*; (**d**) Upper and reverse sides of cultures; (**e**) Conidiomata pycnidial; (**f**) Conidiogenous cells and conidia; (**g**) Conidia; (**h**) Longitudinal section through conidiomata on cotton blue. Scale bars: (**c**) = 250 μm, (**e**) = 250 μm, (**f**) = 10 μm, (**g**) = 5 μm, (**h**) = 100 μm.

*Sinodiscula camellicola* S. Y. Zhao, M. J. Guo, and C. L. Hou, sp. nov.—MycoBank MB851775; Figure 2.

Diagnosis: The new species is similar to *Sinodiscula theae-sinensis*, but differs by the scattered and dark-blown conidiomata with slight raising above the surface of the host tissue at maturity, the bigger conidiomata pycnidial, and the L/W ratio of conidia.

Holotype: China. Anhui Province, Jingde County, Wuguiling, 30.2503 N; 118.3519 E, alt. ca. 392 m, on leaves of *Camellia sinensis*, May 2018, C.L. Hou and Q.T. Wang, living culture CNUCC 107-4-1.

Etymology: Referring to the host plant, *Camellia sinensis*.

On leaves of Camellia sinensis: Conidiomata scattered, round to elliptical or slightly irregular, 160–270 μm diam., dark brown, slight raising above the surface of the host tissue at maturity, opening by an ostiole to liberate the conidia. In the vertical section, conidiomata intraepidermal.

On PDA: Vegetative hyphae 2–5 μm, hyaline, smooth-walled, septate, branched. Conidiomata and Conidiophores formed on a cushion of angular brown cells. Without setae. Conidiomata pycnidial, globular, scattered, immersed in the medium, forming a chamber, erumpent, fuscous to black, 730–1380 μm diam., yellowish to cream conidial drops exuding from the ostioles. Conidiophores acropleurogenous, branched or sympodially branched, cylindrical, aseptate. Conidiogenous cells phialidic, cylindrical, straight or slightly curved, crowded, terminal, slightly tapering toward apex, 7–20 μm × 0.7–2.0 μm, opening 0.4–0.9 μm diam. Conidia small, hyaline, aseptate, smooth-walled, often biguttulate, fusiform to obovoid, tapered at base or both ends, 4.33–5.96 μm × 1.80–2.66 μm, av. ± SD = 5.07 ± 0.41 μm × 2.12 ± 0.20 μm, L/W ratio = 2.40 (*n* = 30).

Culture characteristics: Colony at first white, covered with medium after 15–20 d, becoming olivaceous after 25–30 days. The colony is flat, felty with a thick texture at the center and marginal area, aerial mycelium unconspicuous. Conidiomata sparse, irregularly distributed over agar surface, yellowish mucous conidia were produced on the colony.

The sexual morph: Undetermined.

Additional specimens examined: CHINA. Anhui Province, Jingde County, Niqiuwu, 30.2530 N; 118.3439 E, alt. ca. 460 m, on leaves of *Camellia sinensis*, May 2018, C.L. Hou and Q.T. Wang, living culture CNUCC 106-1-2, CNUCC 106-2-1, and CNUCC 106-3-3; CHINA. Anhui Province, Jingde County, Wuguiling, 30.2503 N; 118.3519 E, alt. ca. 392 m, on leaves of *Camellia sinensis*, May 2018, C.L. Hou and Q.T. Wang, living culture CNUCC 107-3-2; CHINA. Anhui Province, Jingde County, Wuguiling, 30.2504 N; 118.3520 E, alt. ca. 390 m, on leaves of *Camellia sinensis*, May 2018, C.L. Hou and Q.T. Wang, living culture CNUCC 108-2-1; CHINA. Anhui Province, Jingde County, Niqiuwu, 30.2532 N; 118.3440 E, alt. ca. 465 m, on leaves of *Camellia sinensis*, May 2018, C.L. Hou and Q.T. Wang, living culture CNUCC 110-3-3; CHINA. Anhui Province, Jingde County, Yunle, 30.2538 N; 118.3520 E, alt. ca. 430 m, on leaves of *Camellia sinensis*, May 2018, C.L. Hou and Q.T. Wang, living culture CNUCC 111-1-3; CHINA. Anhui Province, Jingde County, Niqiuwu, 30.2533 N; 118.3439 E, alt. ca. 464 m, on leaves of *Camellia sinensis*, May 2018, C.L. Hou and Q.T. Wang, living culture CNUCC 112-2-1, CNUCC 112-2-4, and CNUCC 112-3-3; CHINA. Anhui Province, Jingde County, Niqiuwu, 30.2532 N; 118.3432 E, alt. ca. 432 m, on leaves of *Camellia sinensis*, May 2018, C.L. Hou and Q.T. Wang, living culture CNUCC 114-2-3; CHINA. Anhui Province, Jingde County, Niqiuwu, 30.2522 N; 118.3449 E, alt. ca. 413 m, on leaves of *Camellia sinensis*, May 2018, C.L. Hou and Q.T. Wang, living culture CNUCC 117-1-3 and CNUCC 117-4-4; CHINA. Hubei Province, Yichang City, Muyu Town, 31.4620 N; 110.3980 E, alt. ca. 950 m, on leaves of *Camellia sinensis*, May 2019, C.L. Hou and Q.T. Wang, living culture CNUCC 345-2-3; CHINA. Anhui Province, Jingde County, Yunle, 30.2537 N; 118.3640 E, alt. ca. 445 m, on leaves of *Camellia sinensis*, May 2018, C.L. Hou and Q.T. Wang, living culture CNUCC 1297-2-1.

In the ITS-nrLSU-mtSSU rDNA phylogenetic tree, the molecular sequences of *Sinodiscula camellicola* closely related to the type species *Sinodiscula theae-sinensis*. Morphologically, this new species is similar to *S. theae-sinensis*, but differs by the scattered and dark-blown conidiomata with slight raising above the surface of the host tissue at maturity, the bigger conidiomata pycnidial (240–630 μm diam. vs. 730–1380 μm diam.), and the L/W ratio of conidia (2.20 vs. 2.40). In addition, *S. camellicola* is distinctly separated from *S. theae-sinensis* in gene sequences analysis, the ITS gene shown 86–91% identify, the nrLSU gene shown 93–94% identify.

**Figure 3:**
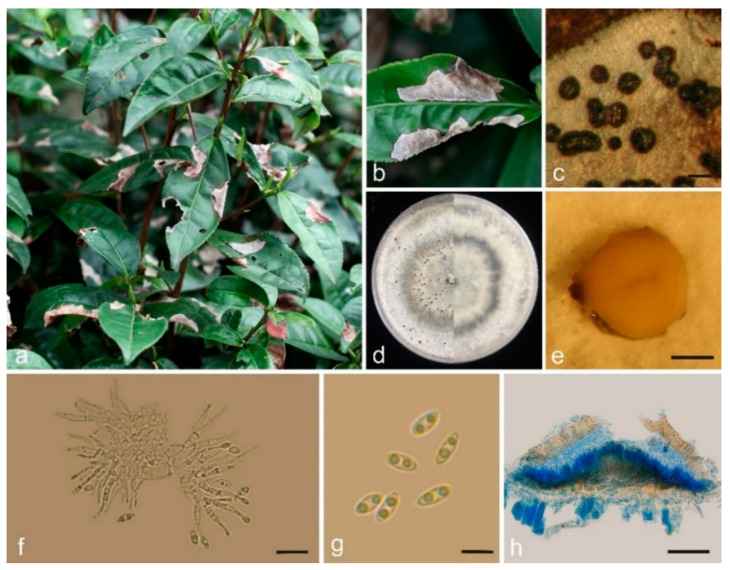
Morphology of *Sinodiscula theae-sinensis* collected from *Camellia sinensis*: (**a**) *Sinodiscula theae-sinensis* causes a large number of fallen leaves of *Camellia sinensis*; (**b**) Lesion on *Camellia sinensis*; (**c**) Habit of conidiomata on leaf of *Camellia sinensis*; (**d**) Upper and reverse sides of cultures; (**e**) Conidiomata pycnidial; (**f**) Conidiogenous cells and conidia; (**g**) Conidia; (**h**) Longitudinal section through conidiomata on cotton blue. Scale bars: (**c**) = 250 μm, (**e**) = 500 μm, (**f**) = 10 μm, (**g**) = 5 μm, (**h**) = 50 μm.

*Sinodiscula theae-sinensis* (I. Miyake) M. J. Guo and C. L. Hou, comb. nov.—MycoBank MB851776; Figure 3.

=*Discula theae-sinensis* (I. Miyake) Moriwaki & Toy. Sato, J. Gen. Pl. Path. 75(5): 359 (2009).

=*Gloeosporium theae-sinensis* I. Miyake, Bot. Mag., Tokyo 21: 44 (1907).

Type. MAFF 752003 (lectotype), isolated from *C. sinensis*, Shiga Pref., Japan, 1984, collected by M. Oniki [50].

Specimen examined. CHINA. Anhui Province, Jingde County, Yunle, 30.2536 N; 118.3602 E, alt. ca. 417 m, on leaves of *Camellia sinensis*, Apr 2017, C.L. Hou and Q.T. Wang, living culture CNUCC 98B-1-2 and CNUCC 98B-2-2; CHINA. Anhui Province, Jingde County, Yunle, 30.2620 N; 118.3580 E, alt. ca. 463 m, on leaves of *Camellia sinensis*, Apr 2017, C.L. Hou and Q.T. Wang, living culture CNUCC 100B-1-3 and CNUCC 100B-3-1; CHINA. Anhui Province, Jingde County, Niqiuwu, 30.2532 N; 118.3440 E, alt. ca. 465 m, on leaves of *Camellia sinensis*, May 2018, C.L. Hou and Q.T. Wang, living culture CNUCC 110-4-2; CHINA. Anhui Province, Jingde County, Yunle, 30.2538 N; 118.3520 E, alt. ca. 430 m, on leaves of *Camellia sinensis*, May 2018, C.L. Hou and Q.T. Wang, living culture CNUCC 111-2-3; CHINA. Zhejiang Province, Wenzhou City, Rui’an City, Hongshuang Forest, 27.7885 N; 120.6580 E, alt. ca. 635 m, on leaves of *Camellia sinensis*, Nov 2018, C.L. Hou and Q.T. Wang, living culture CNUCC 269B-1-1; CHINA. Anhui Province, Jingde County, Yunle, 30.2537 N; 118.3640 E, alt. ca. 445 m, on leaves of *Camellia sinensis*, May 2018, C.L. Hou and Q.T. Wang, living culture CNUCC 1297-2-2, CNUCC 1297-3-1, CNUCC 1297-4-1, and CNUCC 1297-4-4; CHINA. Zhejiang Province, Changshan County, Quzhou, Changshan Oil Tea Park, 29.0350 N; 118.3614 E, alt. ca. 140 m, on leaves of *Camellia sinensis*, May 2023, M.J. Guo, L. Zhuo, and C.L. Hou, living culture CNUCC 1887-3-1; CHINA. Anhui Province, Jingde County, Houjiazhuang, 30.2514 N; 118.3548 E, alt. ca. 335 m, on leaves of *Camellia sinensis*, May 2023, M.J. Guo, L. Zhuo, and C.L. Hou, living culture CNUCC 1900-4-3, 1900-7-3; CHINA. Anhui Province, Jingde County, Houjiazhuang, 30.2504 N; 118.3522 E, alt. ca. 352 m, on leaves of *Camellia sinensis*, May 2023, M.J. Guo, L. Zhuo, and C.L. Hou, living culture CNUCC 1914-2-2, 1914-3-3.

On leaves of Camellia sinensis: Conidiomata scattered or coalesced, round to elliptical or slightly irregular, 130–260 μm diam., black, strongly raising above the surface of the host tissue at maturity, opening by an ostiole to liberate the conidia. In the vertical section, conidiomata intraepidermal.

On PDA: Vegetative hyphae 2–5 μm, hyaline, smooth-walled, septate, branched. Conidiomata and Conidiophores formed on a cushion of angular brown cells. Without setae. Conidiomata pycnidial, globular, scattered, immersed in the medium, forming a chamber, erumpent, fuscous to black, 240–630 μm diam., yellowish to cream conidial drops exuding from the ostioles. Conidiophores acropleurogenous, branched or sympodially branched, cylindrical, aseptate. Conidiogenous cells phialidic, cylindrical, straight or slightly curved, crowded, terminal, slightly tapering toward apex, 10–25 μm × 1–2.5 μm, opening 0.5–1 μm diam. Conidia small, hyaline, aseptate, smooth-walled, often biguttulate, fusiform to obovoid, tapered at base or both ends, 4.15–5.39 μm × 1.20–2.73 μm, av. ± SD = 4.72 ± 0.34 × 2.19 μm ± 0.25 μm, L/W ratio = 2.20 (*n* = 30).

Culture characteristics: Colony at first white, covered with medium after 15–20 d, becoming olivaceous after 25–30 days. The colony is flat, felty with a thick texture at the center and marginal area, aerial mycelium unconspicuous. Conidiomata sparse, irregularly distributed over agar surface, yellowish mucous conidia were produced on the colony.

The sexual morph: Undetermined.

The multi-locus gene analysis indicates that the sequences of the type strain MAFF 752003 described as *Discula theae-sinensis* clustered with other 16 strains in one subclade with high support values (MPB = 100, MLP = 100, PP = 1.00), and not clustered with *Apiognomonia veneta* (Sacc. and Speg.) Höhn., the teleomorph of *Discula nervisequa* (Fuckel) M. Morelet, the type species of *Discula*. Moreover, the morphological observation showed that the strains in this subclade are consistent in morphology. Therefore, we propose a new combination for the present fungus as follows, *Sinodiscula theae-sinensis*.

### 3.3. Pathogenicity Tests

**Figure 4:**
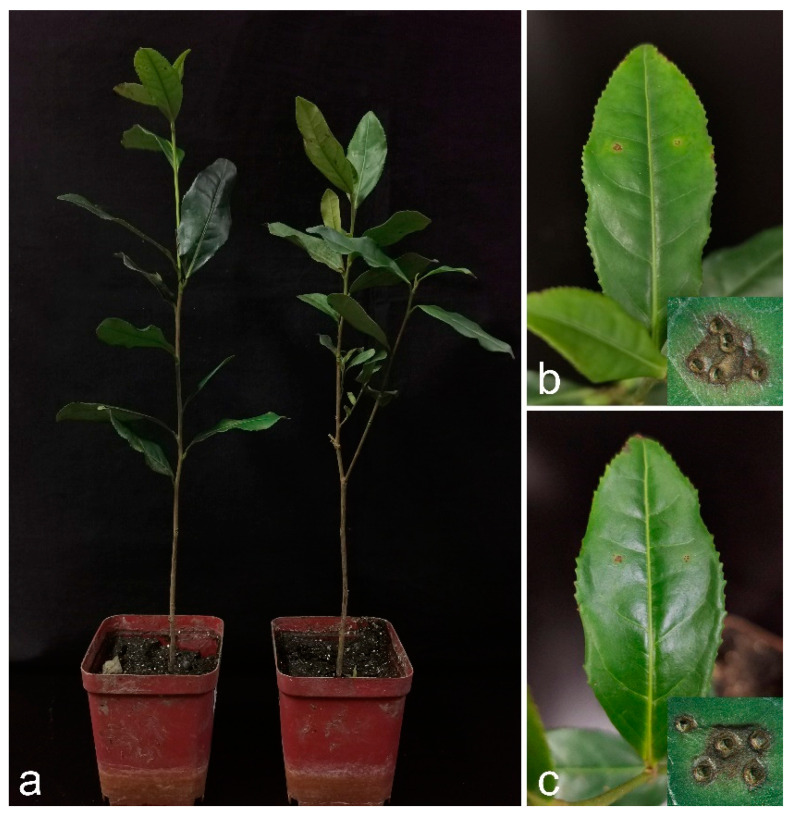
Pathogenicity of *Sinodiscula theae-sinensis* and *Sinodiscula camellicola*. CNUCC 1914-2-2 (*Sinodiscula theae-sinensis*) and CNUCC 107-4-1 (*Sinodiscula camellicola*) were tested on the leaves of *Camellia sinensis:* (**a**) The left is the pathogenicity test of *Sinodiscula theae-sinensis*, the right is the pathogenicity test of *Sinodiscula camellicola*; (**b**) The symptom caused by *Sinodiscula theae-sinensis* after seven days; (**c**) The symptom caused by *Sinodiscula camellicola* after seven days.

For each species of *Sinodiscula*, a representative isolate was selected for the pathogenicity test (CNUCC 1914-2-2 from *Sinodiscula theae-sinensis*, CNUCC 107-4-1 from *Sinodiscula camellicola*). Two isolates of *Sinodiscula* were pathogenic, and the inoculated tea leaves showed lesions similar to the previous symptoms that were observed naturally; nevertheless, the controls remained healthy 7 days after inoculation. Infection occurred from the wound, gradually forming significantly dark lesions on the tea leaf surface (Figure 4). The mean spot size of infected leaves and the incidence of infection are shown in Table 3. The fungi were re-isolated from the lesions and cultured on PDA to verify Koch’s postulates.

## 4. Discussion

In this study, fresh collections of diseased specimens, pure cultures, and multi-locus phylogenetic analysis were used to address the taxonomic and phylogenetic challenges related to the causal fungi of tea anthracnose in China, contributing toward a better understanding of the causal fungi of tea anthracnose in China, and providing clear pathogen information for the further evaluation of the disease control strategies.

As research has progressed, the tea anthracnose pathogen *Gloeosporium theae-sinensis* has undergone several taxonomic changes and has been successively transferred to different genera, namely, *Colletotrichum* and *Discula* [9,14]. However, the morphology of *Gloeosporium theae-sinensis* is characterized by the small conidia, which are much smaller than those of any other species of *Colletotrichum* [13,50]. And as shown in the study of Moriwaki and Sato [9], the conidiogenous cells of the strains of *Gloeosporium theae-sinensis* examined were ampoule-to-tenpin-shaped, like those of the type species of A*piognomonia veneta* (Sacc. and Speg.) Höhn., the teleomorph of *Discula nervisequa* (Fuckel) M. Morelet (*Gnomoniaceae*, *Diaporthales*, *Ascomycota*), rather than a cylindrical shape as in *Colletotrichum* spp. [51,52]. Phylogenetically, the strains isolated from the lesion of anthracnose of tea indeed fell in the same clade of Diaporthalean fungi with high supports, but did not form a clade with any species in this family [9]. Therefore, Moriwaki and Sato suggested that this fungus should belong to the genus *Discula* [9]. In this study, the morphological and molecular phylogenetic analyses indicate that the isolates of the causal fungus of the tea anthracnose belong to *Melanconiellaceae*, but cannot be classified within any existing genus of *Melanconiellaceae*. Therefore, a novel genus *Sinodiscula* is proposed in this study, typified by the new combination *Sinodiscula theae-sinensis*, and a new species *Sinodiscula camellicola* is also described. Indeed, these two species exhibit remarkable morphological similarities and lack significant differences in terms of pathogenicity, which presents challenges in their differentiation. However, there is a noticeable molecular distinction between *Sinodiscula camellicola* and *Sinodiscula theae-sinensis*, underscoring the importance of molecular markers in distinguishing between these two species. Further comparative research using genomic approaches in order to gain a more comprehensive understanding of these species should be conducted.

The use of the name “tea anthracnose” has long been controversial, because the disease of tea caused by *Discula theae-sinensis*, a synonym of *Gloeosporium theae-sinensis*, is commonly referred to as “tea anthracnose” [8,9,10,53,54,55]. However, the disease of tea caused by *Colletotrichum* spp. is also referred to as “tea anthracnose” [56,57,58,59,60]. It is noteworthy that the phytopathogenic fungi causing “tea anthracnose” do not belong to the same family or order. While the disease of tea caused by *Colletotrichum camelliae* Massee is known as “tea cloud leaf blight” in China, this name is not widely used [11,61,62,63]. Phylogenetically, the species of *Colletotrichum* spp. that cause “tea anthracnose” are distantly related to *Discula theae-sinensis* [64]. However, the symptoms of the disease they cause on the leaves of tea are very similar. The disease caused by these phytopathogenic fungi primarily affects mature leaves and typically begins at the leaf edge or tip. Initially, they produce dark green or yellowish-brown watery spots that later expand along the leaf veins, forming irregular-shaped spots. These spots gradually turn brown or reddish-brown and eventually become greyish-white. The edges of the spots have a yellowish-brown line and are clearly distinguishable from the healthy part of the leaf. The front of the spot is densely covered with numerous small black conidiomata [11,12,58]. The visual similarity of disease symptoms caused by these pathogens makes it challenging to differentiate them with the naked eye, which contributes to the confusion surrounding their identification. The study of Li et al. [18] revealed that *Discula theae-sinensis* is the predominant species in tea leaves, serving as the primary causative agent of tea plant anthracnose. However, there are numerous studies on “tea anthracnose” caused by *Colletotrichum* spp., so we suggest that the tea disease caused by *Colletotrichum* spp. is referred to as “tea anthracnose” and the tea disease caused by *Discula theae-sinensis* as “the tea leaf blight”, in order to differentiate between the two diseases.

## 5. Conclusions

In this study, dozens of specimens and strains of *Discula theae-sinensis*, collected and isolated from diseased leaves of tea in Anhui, Hubei, and Zhejiang Provinces, China, were investigated. The phylogeny and taxonomy of *Discula theae-sinensis* were revised through phylogenetic analyses, morphological characteristics, and pathogenicity tests. A novel genus *Sinodiscula* is proposed to accommodate the pathogens of tea anthracnose, typified by the new combination *Sinodiscula theae-sinensis*, and a new species, *Sinodiscula camellicola,* is included. Additionally, the controversial use of the name “tea anthracnose” was discussed, suggesting that the tea disease caused by *Sinodiscula* should be referred to as “the tea leaf blight”. Accurate diagnosis of plant diseases is crucial for effective disease management, and further research and verification of disease control methods are necessary in the near future.

## Figures and Tables

**Table 1:** Primers used in this study, with sequences and sources.

Gene ^a^	Primer	Sequence (5′–3′)	Expected Amplicon Size (bp)
ITS	ITS1f	CTTGGTCATTTAGAGGAAGTAA	600
	ITS4	TCCTCCGCTTATTGATATGC	
nrLSU	LR5	TCCTGAGGGAAAGTTCG	1200
	LR0R	ACCCGCTGAACTTAAGC	
*rpb2*	5f	GAYGAYMGWGATCAYTTYGG	1200
	7cR	CCCATRGCTTGYTTRCCCAT	
*tef1*	728f	CATCGAGAAGTTCGAGAAGG	600
	EF2	GGARGTACCAGTSATCATGTT	

^a^ ITS, the internal transcribed spacer regions and intervening 5.8S nrDNA; nrLSU, the present study uses a combined taxonomic approach based on morphology and DNA sequence analyses of the partial 28S nrDNA; *rpb2*, DNA-directed RNA polymerase II second largest subunit; *tef1*, translation elongation factor 1-alpha.

**Table 2:** GenBank accession numbers and culture collection/isolate information for the molecular analysis of *Diaporthales*. Information written in **bold** refers to sequences generated in the context of the present study.

Species	Culture Collection/Isolate	ITS	nrLSU	*rpb2*	*tef1*
*Apiognomonia veneta*	CBS 897.79	-	EU255195	EU219259	EU221910
*Apiosporopsis carpinea*	CBS 771.79	AF277130	-	-	-
*Apoharknessia insueta*	CBS 111377	AY720814	JQ706083	-	MN271820
*Apoharknessia insueta*	CBS 114575	MN172370	MN172402	-	MN271821
*Asterosporium asterospoermum*	MFLU 15–3555	MF190062	-	-	-
*Auratiopycnidiella tristaniopsis*	CBS 132180	JQ685522	JQ685516	-	MN271825
*Auratiopycnidiella tristaniopsis*	CPC 16371	MN172374	MN172405	-	MN271826
*Ceratosphaeria aquatica*	MFLU 18–2323	MK835812	MK828612	MN156509	MN194065
*Chapeckia nigrospora*	AR 3809	EU683068	-	-	-
*Chrysoporthe australafricana*	CBS 112916	AY194097	AF292041	-	MN271832
*Chrysoporthe cubensis*	CBS 118654	MN172378	DQ368773	-	MN271834
*Coneilla peruensis*	CBS 110394	KJ710441	KJ710463	KX833499	KX833695
*Coniella eucalyptorum*	CBS 112640	AY339290	AY339338	KX833452	KX833637
*Coryneum gigasporum*	CFCC 52319	MH683557	MH683565	-	-
*Coryneum umbonatum*	D201	MH674329	MH674329	MH674333	MH674337
*Cryphonectria parasitica*	ATCC 38755	NG_027589	AY141856	DQ862017	EU222014
*Cryphonectria parasitica*	ATCC 48198	JN940858	JN942325	-	-
*Cryphonectria parasitica*	CFCC 52150	MH514021	MG866018	-	MN271848
*Cytospora chrysosperma*	CFCC 89982	KP310805	KP281261	KU710952	KP310848
*Cytospora eleagni*	CFCC 89633	KF765693	KF765677	KU710956	KU710919
*Cytospora viridistroma*	CBS 202.36	MN172388	MN172408	-	MN271853
*Dendrostoma osmanthi*	CFCC 52106	MG682013	MG682073	MG682033	MG682053
*Diaporthe citri*	AR 3405	MT378365	KC843311	MT383081	KC843071
*Diaporthe eres*	CBS 138594	MT378367	KJ210529	MT383083	KJ210550
*Diaporthe helianthi*	CBS 592.81	MT378370	NR_103698	-	KC343841
*Diaporthe novem*	AR 4855	MT378366	MT378351	MT383082	MT383100
*Diaporthella cryptica*	CBS 140348	MN172390	MN172409	MN271800	MN271854
*Diaporthosporella cercidicola*	CFCC 51994	KY852515	KY852492	-	MN271855
*Diaporthostoma machili*	CFCC 52101	MG682021	MG682081	MG682041	MG682061
*Diaporthostoma machili*	CFCC 52100	MG682020	MG682080	MG682040	MG682060
*Dicarpella* sp.	NY7452a	HM855225	-	-	-
*Discula betulina*	AR 4173	EU254757	-	-	-
*Discula campestris*	CBS 32875	EU199185	MH872664	EU199143	-
*Discula destructiva*	CBS 109771	-	-	EU199144	JQ414137
*Discula quercina*	CBS 115013	AY853196	-	-	-
*Dwiroopa lythri*	CBS 109755	MN172389	MN172410	MN271801	MN271859
*Dwiroopa punicae*	CBS 143163	MK510686	MK510676	MK510692	-
*Erythrogloeum hymenaeae*	CBS 132185	JQ685525	JQ685519	-	-
*Gnomoniopsis comari*	CBS 806.79	EU255114	EU254821	-	GU320810
*Gnomoniopsis fragariae*	CBS 121226	EU255115	EU254824	EU219250	EU221961
*Gnomoniopsis fragariae*	DMW 63	MT378357	MT378343	MT383072	MT383089
*Gnomoniopsis fragariae*	DMW 61	MT378358	MT378344	MT383073	MT383090
*Gnomoniopsis fragariae*	VPRI 15547	MT378359	MT378345	MT383087	MT383091
*Gnomoniopsis fragariae*	CBS 275.51	MH868373	EU254829	MT383088	MT383092
*Gnomoniopsis fragariae*	CBS 208.34	EU255116	EU254826	EU219284	EU221968
*Gnomoniopsis tormentillae*	CBS 904.79	EU255133	EU254856	-	GU320795
*Greeneria uvicola*	AU01	JN547720	-	-	-
*Greeneria uvicola*	OH35	AF362570	-	-	-
*Harknessia gibbosa*	CBS 120033	EF110615	EF110615	-	MN271868
*Harknessia ipereniae*	CBS 120030	EF110614	EF110614	-	MN271870
*Involutiscutellula rubra*	MUCC 2304	MG591995	MG591901	MG976478	MG592088
*Involutiscutellula rubra*	CBS 192.71	MG591993	MG591899	MG976476	MG592086
*Juglanconis juglandina*	CBS 121083	KY427148	KY427148	KY427198	KY427217
*Juglanconis oblonga*	MAFF 410216	KY427153	KY427153	KY427203	KY427222
*Juglanconis pterocaryae*	MAFF 410079	KY427155	KY427155	KY427205	KY427224
*Lamproconium desmazieri*	MFLUCC 15–0870	KX430135	KX430134	MF377605	MF377591
*Lamproconium desmazieri*	MFLUCC 15–0872	KX430139	KX430138	-	MF377593
*Macrohilum eucalypti*	CBS 140063	NG_058183	NR_154184	MN271810	-
*Macrohilum eucalypti*	CPC 10945	DQ195793	DQ195781	-	-
*Mastigosporella anisophylleae*	CBS 136421	KF777221	KF779492	-	MN271892
*Mastigosporella pigmentata*	VIC44383	MG587928	MG587929	-	-
*Mazzantia galii*	CBS 125529	MH875041	MH863563	-	MT383101
*Melanconiella chrysodiscosporina*	CBS 125597	MH875191	MH863730	-	-
*Melanconiella cornuta*	CFCC 51990	MF360006	MF360008	MF360002	MF360004
*Melanconiella cornuta*	CFCC 51991	MF360007	MF360009	MF360003	MF360005
*Melanconiella elegans*	AR 3830	JQ926264	JQ926264	JQ926335	JQ926401
*Melanconiella ellisii*	BPI 878343	JQ926271	JQ926271	JQ926339	JQ926406
*Melanconiella spodiaea*	AR 3457	AF408369	MT378352	MT383074	MT383093
*Melanconiella spodiaea*	AR 3462	AF408370	MT378353	MT383075	MT383094
*Melanconis betulae*	CFCC 50471	KT732971	KT732952	KT732984	KT733001
*Melanconis itoana*	CFCC 50474	KT732974	KT732955	KT732987	KT733004
*Melanconis stilbostoma*	CFCC 50475	KT732975	KT732956	KT732988	KT733005
*Microascospora rubi*	MFLU 15–1112	MF190099	MF190154	MF377611	MF377582
*Microascospora rubi*	MFLU 17–0883	MF190098	MF190153	-	MF377581
*Neocryphonectria carpini*	CFCC 53027	MN172396	MN172413	-	-
*Neocryphonectria chinensis*	CFCC 53025	MN172397	MN172414	MN271812	MN271893
*Neopseudomelanconis castaneae*	CFCC 52787	MH469164	MH469162	-	-
*Oblongisporothyrium castanopsidis*	CBS 189.71	MG591943	MG591850	-	MG592038
*Oblongisporothyrium castanopsidis*	CBS 124732	MG591942	MG591849	MG976453	MG592037
*Ophiognomonia rosae*	DMW 108	MT378355	JF514851	MT383086	JF514824
*Ophiognomonia rosae*	CBS 851.79	MT378356	EU254930	MT383071	JQ414153
*Paragnomonia fragariae*	F129	MK524447	MK524430	-	MK524466
*Paragnomonia fragariae*	GF300	MT378368	-	MT383084	MT383102
*Paragnomonia fragariae*	GF301	MT378369	-	MT383085	MT383103
*Paraphomopsis obscurans*	M1261	MT378360	MT378346	MT383076	MT383095
*Paraphomopsis obscurans*	CBS 143829	MT378361	MT378347	MT383077	MT383096
*Paraphomopsis obscurans*	M1259	MT378362	MT378348	MT383078	MT383097
*Paraphomopsis obscurans*	M1333	MT378363	MT378349	MT383079	MT383098
*Paraphomopsis obscurans*	M1278/DS055	MT378364	MT378350	MT383080	MT383099
*Paraphomopsis obscurans*	strain 1–1	-	HM854850	-	-
*Paraphomopsis obscurans*	strain 1–3	-	HM854852	-	-
*Paraphomopsis obscurans*	strain 12	-	HM854849	-	-
*Paratubakia subglobosa*	CBS 193.71	MG592009	MG591914	MG976490	MG592103
*Paratubakia subglobosoides*	MUCC 2293	MG592010	MG591915	MG976491	MG592104
*Phaeoappendicospora thailandensis*	MFLUCC 13–0161	MF190102	MF190157	-	-
*Prosopidicola mexicana*	CBS 113529	KX228354	AY720709	-	-
*Prosopidicola albizziae*	CBS 141298	KX228325	KX228274	-	-
*Pseudomelanconis caryae*	CFCC 52110	MG682022	MG682082	MG682042	MG682062
*Pseudoplagiostoma eucalypti*	CPC 14161	GU973604	GU973510	-	GU973540
*Pseudoplagiostoma oldii*	CBS 115722	GU973610	GU973535	-	GU973565
*Pyricularia grisea*	CG-4	JX134683	JX134671	-	JX134697
*Racheliella wingfieldiana*	CPC 13806	MG592006	MG591911	MG976487	MG592100
*Septomelanconiella thailandica*	MFLUCC 18-0518	MH727706	MH727705	MH752072	-
*Sillia karstenii*	MFLU 16–2864	KY523500	KY523482	KY501636	-
***Sinodiscula camellicola***	**CNUCC 106-1-2**	**PP150390**	**PP149025**	**PP174323**	**PP156920**
***Sinodiscula camellicola***	**CNUCC 106-2-1**	**PP150391**	**PP149026**	**PP174324**	**PP156921**
***Sinodiscula camellicola***	**CNUCC 106-3-3**	**PP150392**	**PP149027**	**PP174325**	**PP156922**
***Sinodiscula camellicola***	**CNUCC 107-3-2**	**PP150393**	**PP149028**	**PP174326**	**PP156923**
***Sinodiscula camellicola***	**CNUCC 107-4-1**	**PP150394**	**PP149029**	**PP174327**	**PP156924**
***Sinodiscula camellicola***	**CNUCC 108-2-1**	**PP150395**	**PP149030**	**PP174328**	**PP156925**
***Sinodiscula camellicola***	**CNUCC 110-2-3**	**PP150396**	**PP149031**	**PP174329**	**PP156926**
***Sinodiscula camellicola***	**CNUCC 111-1-3**	**PP150397**	**PP149032**	**PP174330**	**PP156927**
***Sinodiscula camellicola***	**CNUCC 112-2-1**	**PP150398**	**PP149033**	**PP174331**	**PP156928**
***Sinodiscula camellicola***	**CNUCC 112-2-4**	**PP150399**	**PP149034**	**PP174332**	**PP156929**
***Sinodiscula camellicola***	**CNUCC 112-3-3**	**PP150400**	**PP149035**	**PP174333**	**PP156930**
***Sinodiscula camellicola***	**CNUCC 114-2-3**	**PP150401**	**PP149036**	**PP174334**	**PP156931**
***Sinodiscula camellicola***	**CNUCC 117-1-3**	**PP150402**	**PP149037**	**PP174335**	**PP156932**
***Sinodiscula camellicola***	**CNUCC 117-4-4**	**PP150403**	**PP149038**	**PP174336**	**PP156933**
***Sinodiscula camellicola***	**CNUCC 345-2-3**	**PP150404**	**PP149039**	**PP174337**	**PP156934**
***Sinodiscula camellicola***	**CNUCC 1297-2-1**	**PP150405**	**PP149040**	**PP174338**	**PP156935**
***Sinodiscula theae-sinensis***	**MAFF 238240**	**AB511919**	-	-	-
***Sinodiscula theae-sinensis***	**MAFF 238241**	**AB511920**	-	-	-
***Sinodiscula theae-sinensis***	**MAFF 238242**	**AB511921**	-	-	-
***Sinodiscula theae-sinensis***	**MAFF 238243**	**AB511922**	-	-	-
***Sinodiscula theae-sinensis***	**MAFF 238244**	**AB511923**	-	-	-
***Sinodiscula theae-sinensis***	**MAFF 752003**	**PP150406**	**PP149041**	**PP174339**	**PP156936**
***Sinodiscula theae-sinensis***	**CNUCC 98B-1-2**	**PP150407**	**PP149042**	**PP174340**	**PP156937**
***Sinodiscula theae-sinensis***	**CNUCC 98B-2-2**	**PP150408**	**PP149043**	**PP174341**	**PP156938**
***Sinodiscula theae-sinensis***	**CNUCC 100B-1-3**	**PP150409**	**PP149044**	**PP174342**	**PP156939**
***Sinodiscula theae-sinensis***	**CNUCC 100B-3-1**	**PP150410**	**PP149045**	**PP174343**	**PP156940**
***Sinodiscula theae-sinensis***	**CNUCC 110-4-2**	**PP150411**	**PP149046**	**PP174344**	**PP156941**
***Sinodiscula theae-sinensis***	**CNUCC 111-2-3**	**PP150412**	**PP149047**	**PP174345**	**PP156942**
***Sinodiscula theae-sinensis***	**CNUCC 269B-1-1**	**PP150413**	**PP149048**	**PP174346**	**PP156943**
***Sinodiscula theae-sinensis***	**CNUCC 1297-2-2**	**PP150414**	**PP149049**	**PP174347**	**PP156944**
***Sinodiscula theae-sinensis***	**CNUCC 1297-3-1**	**PP150415**	**PP149050**	**PP174348**	**PP156945**
***Sinodiscula theae-sinensis***	**CNUCC 1297-4-1**	**PP150416**	**PP149051**	**PP174349**	**PP156946**
***Sinodiscula theae-sinensis***	**CNUCC 1297-4-4**	**PP150417**	**PP149052**	**PP174350**	**PP156947**
***Sinodiscula theae-sinensis***	**CNUCC 1887-3-1**	**PP150418**	**PP149053**	**PP174351**	**PP156948**
***Sinodiscula theae-sinensis***	**CNUCC 1900-4-3**	**PP150419**	**PP149054**	**PP174352**	**PP156949**
***Sinodiscula theae-sinensis***	**CNUCC 1900-7-3**	**PP150420**	**PP149055**	**PP174353**	**PP156950**
***Sinodiscula theae-sinensis***	**CNUCC 1914-2-2**	**PP150421**	**PP149056**	**PP174354**	**PP156951**
***Sinodiscula theae-sinensis***	**CNUCC 1914-3-3**	**PP150422**	**PP149057**	-	**PP156952**
*Sphaerosporithyrium mexicanum*	CPC 31361	MG591988	MG591894	-	MG592081
*Sphaerosporithyrium mexicanum*	CPC 33021	MG591990	MG591896	MG976473	MG592083
*Stegonsporium acerophilum*	CBS 117025	EU039993	EU039982	KF570173	EU040027
*Stilbospora longicornuta*	CBS 122529	KF570164	KF570164	KF570194	KF570232
*Synnemasporella aculeans*	CFCC 52094	MG682026	MG682086	MG682046	MG682066
*Synnemasporella toxicodendri*	CFCC 52097	MG682029	MG682089	MG682049	MG682069
*Thailandiomyces bisetulosus*	BCC 00018	EF622230	-	-	-
*Tirisporella beccariana*	BCC 38312	JQ655449	-	-	-
*Tubakia dryina*	CBS 114386	JF704188	MG591852	-	MG592040
*Tubakia iowensis*	CBS 129012	MG591971	JF704194	-	MG603576
*Tubakia seoraksanensis*	CBS 127490	KP260499	MG591907	-	MG592094

Abbreviations of the culture collections: ATCC: American Type Culture collection; CMW:FABI fungal culture collection; CBS: CBS-KNAW culture collection, Westerdijk Fungal Biodiversity Institute; MFLU: Mae Fah Luang University Herbarium; MFLUCC: Mae Fah Luang University Culture Collection; CFCC: China Forestry Culture Collection Center; STE-U: culture collection of the Department of Plant Pathology at the University of Stellenbosch; AR, M, DMW: Cultures housed at MNGDBL, USDA-ARS, Beltsville, Maryland; CPC: Culture collection of Pedro Crous, housed at Westerdijk Fungal Biodiversity Institute; MUCC: Murdoch University Culture Collection; BCC: BIOTEC Culture Collection, Bangkok, Thailand; VPRI: Victoria Plant Pathology Herbarium.

**Table 3:** The mean spot size of infected leaves and the incidence of infection.

Species	The Mean Spot Size (av. ± SD/mm)	Incidence
*Sinodiscula theae-sinensis* (CNUCC 1914-2-2)	1.52 ± 0.18	100%
*Sinodiscula camellicola* (CNUCC 107-4-1)	1.21 ± 0.09	88.9%

## Data Availability

Data are contained within the article.
